# Microlaryngeal surgery in severely obese elite vocal performers

**DOI:** 10.1002/ccr3.4680

**Published:** 2021-08-15

**Authors:** Tomohiro Hasegawa, Daigo Komazawa, Yusuke Watanabe

**Affiliations:** ^1^ Tokyo Voice Center International University of health and Welfare Tokyo Japan; ^2^ AKASAKA Voice Health Center Tokyo Japan

**Keywords:** EVP, preoperative simulation, ramp position, severe obese patients

## Abstract

This study showed that microlaryngeal surgery under general anesthesia is feasible for patients with severe obese elite vocal performers if proper simulations are conducted beforehand and the position of the patient and anesthesia is considered.

## Graphical abstract

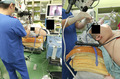

## 1 INTRODUCTION

The management of general anesthesia in obese patients is considered risky. This report describes two cases of obese elite vocal performers who underwent preoperative simulation and successful microlaryngeal surgery under general anesthesia. We believe that microlaryngeal surgery under general anesthesia is feasible with preoperative simulations.

Obesity is rising worldwide, and the obese population with a body mass index (BMI) >30 kg/m^2^ in 2016 is said to have tripled since 1975, constituting 13% of the global population.^1^ The management of general anesthesia in severely obese patients is considered riskier than in non‐obese patients. Therefore, when severely obese patients suffer from vocal fold lesions in Japan, surgery under local anesthesia using an endoscopic fiber is often preferred over microlaryngeal surgery. However, it is quite challenging to perform delicate surgery using an endoscopic fiber under local anesthesia on microvocal lesions. In addition, if the patient is an elite vocal performer (EVP), vocal fold lesion removal under local anesthesia may not improve postoperative voice performance as much as desired. This report describes two cases of microlaryngeal surgery under general anesthesia in severely obese EVPs, including a discussion of the literature. This study was reviewed by the Ethics Committee of our Hospital with Approval Number.

## 2 CASE PRESENTATION

Patient 1 was a 27‐year‐old Japanese male (height, 168 cm; weight, 153 kg; BMI, 54) with a complaint of falsetto disorder. He worked as a pop singer and had a history of diabetes and fatty liver. At the time of his visit to our clinic, he had lost his falsetto after overusing his voice in singing practice a month previously. The patient was diagnosed with bilateral vocal fold polyps and underwent microlaryngeal surgery under general anesthesia.

**FIGURE 1:**
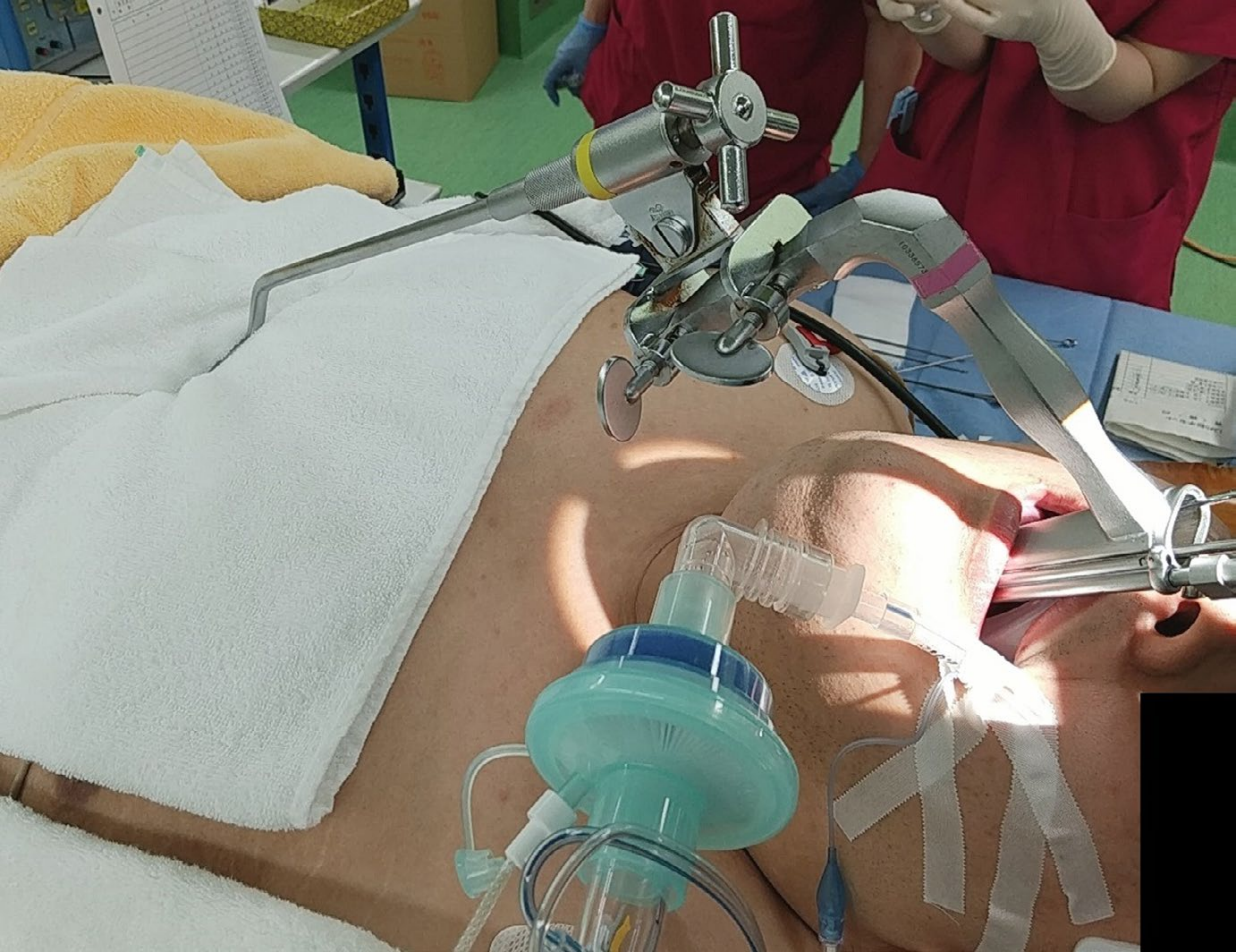
Towel is placed on the chest and the laryngoscope is suspended directly on the towel

**FIGURE 2:**
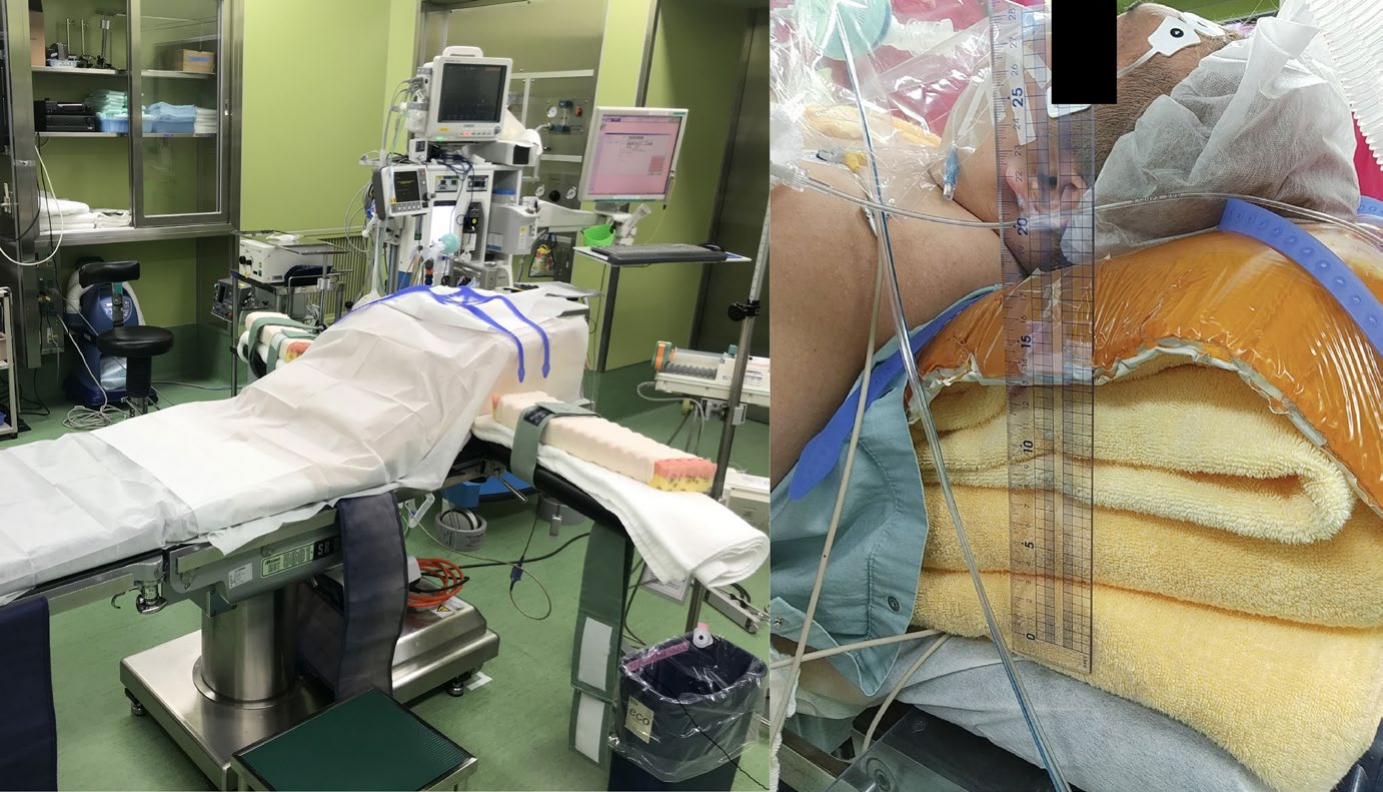
Ramp position for induction of general anesthesia

**FIGURE 3:**
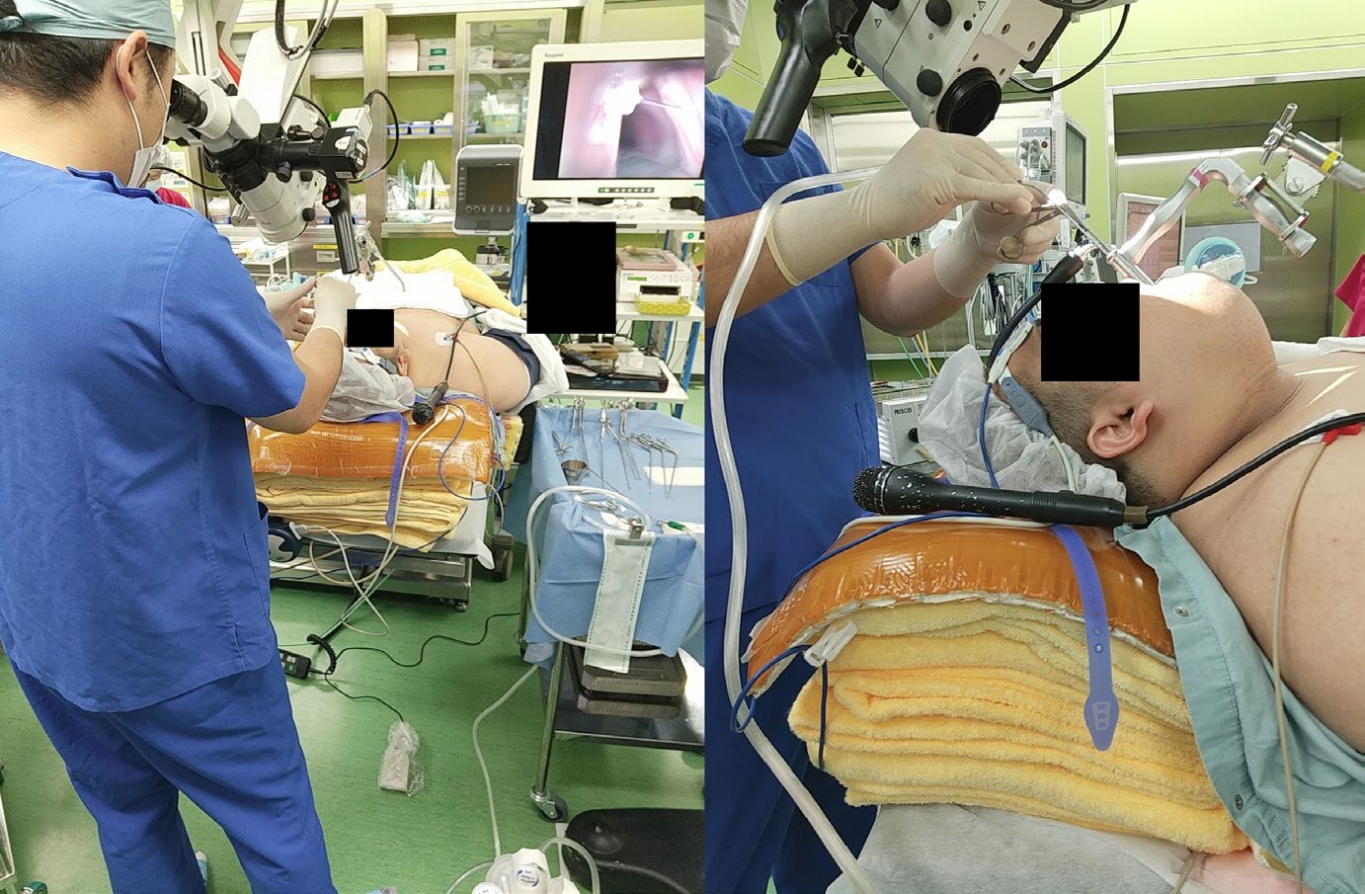
Entire procedure is performed in the upright position due to the ramp position

Before induction of anesthesia, we conducted a preoperative simulation on the anesthesia table with the cooperation of the patient. The patient himself was asked to lie on the operating table in the operating room where microlaryngeal surgery was performed. Due to the thickness of the chest, it was not possible to set up a stand to hold the laryngoscope. Therefore, we chose to place a towel on the chest and the laryngoscope suspension directly on the towel (Figure 1), and we explained the possibility of postoperative chest pain to the patient. The patient was then intubated in the ramp position for induction of general anesthesia (Figure 2). Because of the ramp position, all microlaryngeal surgery procedures, which are usually performed in the sitting position, were performed by the surgeon in the standing position (Figure 3). After a scalpel incision was made at the base of the bilateral vocal fold polyps, the lesions were removed with forceps. The postoperative course was good, and there was no pain in the trunk region, which was compressed by the base of the laryngoscope holder. The operation time was 11 min and the anesthesia time was 69 min, and no major problems were observed in the vital signs during the operation. The patient is currently under outpatient observation, and the head voice disorder is improving. Maximum phonation time (MPT) improved from 20.0 seconds to 37.5 seconds after one month.

Patient 2 was a 58‐year‐old American male (height, 172 cm; weight 143 kg; BMI 48). He had a medical history of postoperative kidney cancer and hypertension. He worked as an R & B singer and had been experiencing falsetto and mixed voice problems while singing for a year. His symptoms did not improve, so he visited his local doctor and was referred to our hospital for surgery. The patient was diagnosed with bilateral vocal fold nodules and underwent microlaryngeal surgery under general anesthesia.

**FIGURE 4:**
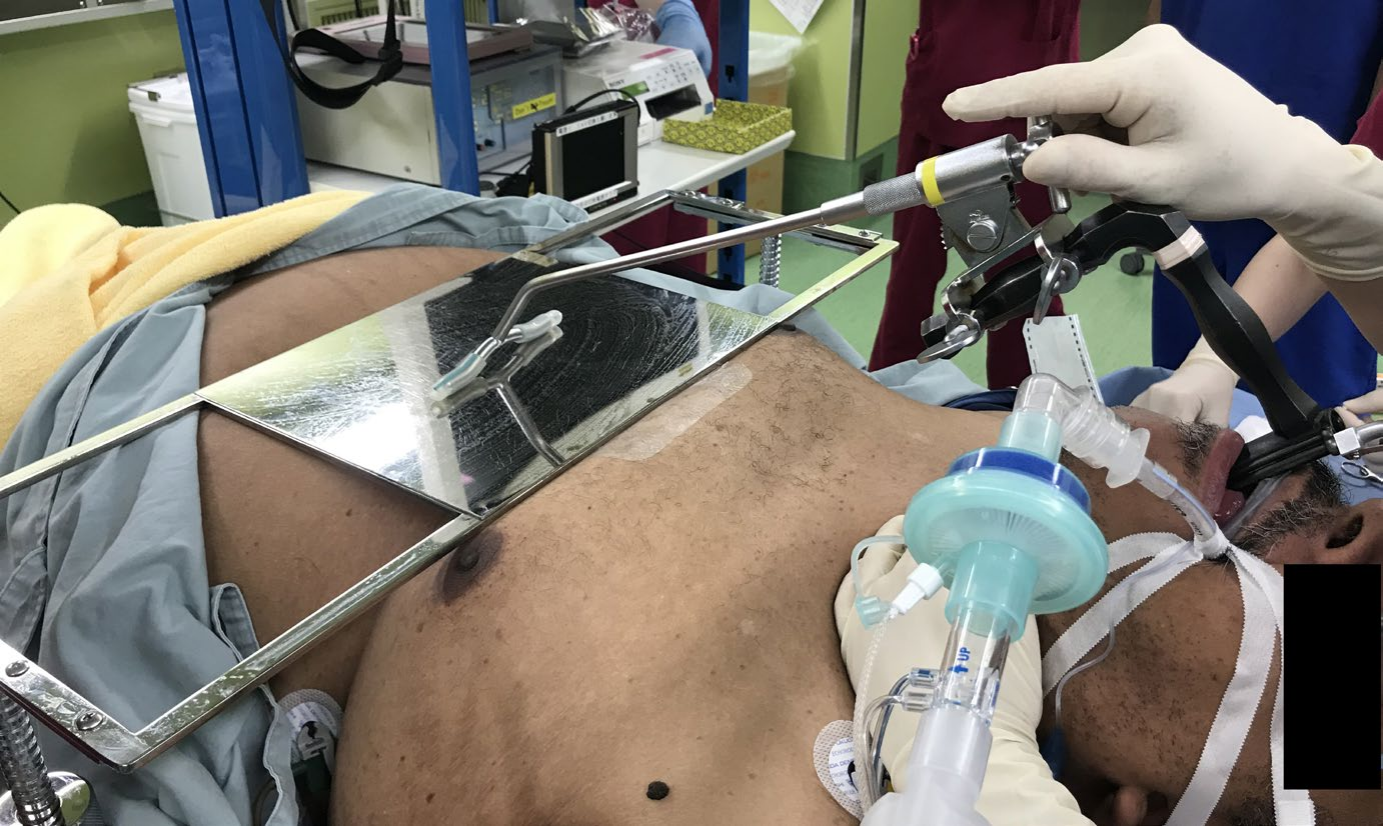
Stand to fix the laryngoscope is placed directly on the patient's chest and a laryngoscope suspension is placed on top of the stand to fix the laryngoscope. The stand is not fixed to the operating table

Before induction of anesthesia, we conducted a preoperative simulation on the anesthesia table with the cooperation of the patient. The thickness of his chest did not allow us to erect the stand to fix the laryngoscope, so we placed the stand directly on the chest. The laryngoscope suspension was placed directly on the stand and fixed in place (Figure 4). The patient was intubated in the ramp position, and anesthesia induction was performed without any problems. A broad basal nodular lesion was found below the vocal cords on both sides and was resected with forceps after a scalpel incision was made at the base. The operation time was 13 min and anesthesia time was 54 min, and no major problems were observed in the vital signs during the operation. The postoperative course was good, and the patient is currently under outpatient observation. MPT improved from 6.0 seconds to 8.5 seconds after one month.

## 3 DISCUSSION

We performed microlaryngeal surgery under general anesthesia in two patients who were severely obese EVPs. Although voice therapy effectively treats vocal fold nodules, the patients themselves expressed a desire for early voice improvement; therefore, we performed microlaryngeal surgery. In both cases, we performed preoperative simulations on an actual anesthesia table in cooperation with anesthesiologists and operating room nurses before surgery. Hence, we found beforehand that the stand could not be fixed to the bed in both cases. In this study, without preoperative simulation, we found that the stand could not be positioned after the induction of anesthesia, which could have unnecessarily prolonged anesthesia and surgery time. Therefore, if possible, we believe that it is necessary to conduct preoperative simulation before the induction of anesthesia for severely obese patients at each facility.

In general, anesthesia for severely obese patients is considered risky. According to the ASA physical status classification system last approved by the ASA House of Delegates on October 15, 2014^2^ (Table 1), severe obesity (BMI >40) is the third of the five levels and is equivalent to chronic obstructive pulmonary disease and moderately reduced cardiac ejection fraction. The ramp position is selected for intubation of obese patients, in which a towel or soft cloth pillow is placed from the shoulder to the neck of the obese patient to maintain a semi‐sitting position (Figure 2). This position is recommended as a good choice for tracheal intubation in such cases.^3^, ^4^, ^5^ However, due to the ramp position, the microlaryngeal surgery, usually performed sitting in a chair, was performed standing (Figure 3). When performed in the standing position, the angle of entry of the scalpel and forceps to the horizontal direction is slightly steeper. Although microlaryngeal surgery deals with microscopic lesions, the technique itself is not affected much.

**TABLE 1:** Current Definitions and ASA‐Approved Examples

ASA PS Classification	Definition	Adult Examples, Including, but not Limited to
ASA I	A normal healthy patient	Healthy, non‐smoking, no or minimal alcohol use
ASA II	A patient with mild systemic disease	Mild diseases only without substantive functional limitations. Current smoker, social alcohol drinker, pregnancy, obesity (30<BMI<40), well‐controlled DM/HTN, mild lung disease
ASA III	A patient with severe systemic disease	Substantive functional limitations; One or more moderate to severe diseases. Poorly controlled DM or HTN, COPD, morbid obesity (BMI ≥40), active hepatitis, alcohol dependence or abuse, implanted pacemaker, moderate reduction of ejection fraction, ESRD undergoing regularly scheduled dialysis, history (>3 months) of MI, CVA, TIA or CAD/stents.
ASA IV	A patient with severe systemic disease that is a constant threat to life	Recent (<3 months) MI, CVA, TIA or CAD/stents, ongoing cardiac ischemia or severe valve dysfunction, severe reduction of ejection fraction, shock, sepsis, DIC, ARD, or ESRD not undergoing regularly scheduled dialysis
ASA V	A moribund patient who is not expected to survive without the operation	Ruptured abdominal/thoracic aneurysm, massive trauma, intracranial bleed with mass effect, ischemic bowel in the face of significant cardiac pathology or multiple organ/system dysfunction
ASA VI	A declared brain‐dead patient whose organs are being removed for donor purposes	–

## 4 CONCLUSION

We believe that microlaryngeal surgery under general anesthesia is feasible in patients with severe obesity, provided that the anesthesiologist and operating room nurse work together to perform simulations beforehand and consider the position and anesthesia.

## CONFLICTS OF INTEREST

There are no conflicts of interest in this paper.

## AUTHOR CONTRIBUTIONS

Author 1: Daigo Komazawa advised me on the surgery. Author 2: Yusuke Watanabe supervised the surgery and research.

## Data Availability

The data that support the findings of this study are available from the corresponding author upon reasonable request.
